# Natural variations at the E433 site in Env-gp120 β20-β21 cooperate with V2 and gp41 mutations to modulate HIV-1 infectivity

**DOI:** 10.3389/fmicb.2026.1877899

**Published:** 2026-09-15

**Authors:** Bao Quoc Le, Khanh Quoc Tran, Naoya Doi, Takaaki Koma, Tomoyuki Kondo, Osamu Kotani, Hironori Sato, Shun Adachi, Akio Adachi, Masaru Yokoyama, Masako Nomaguchi

**Affiliations:** 1 Department of Microbiology, Graduate School of Medicine, Tokushima University, Tokushima, Japan; 2 Division of Interdisciplinary Researches for Medicine and Photonics, Institute of Post-LED Photonics, Tokushima University, Tokushima, Japan; 3 Department of Bioinformatics and Integrative Omics, National Institute of Infectious Diseases, Japan Institute for Health Security, Musashimurayama, Japan; 4 Independent Researcher, Uji, Japan

**Keywords:** adaptive mutations, CD4 interaction, Env, HIV-1, β20-β21 hairpin

## Abstract

HIV-1 infection begins with Env-gp120 binding to the CD4 receptor and CD4-triggered Env conformational changes that enable viral entry. To identify key Env sites that modulate Env-CD4 interactions, we analyzed growth-enhanced adaptive mutants generated through the *in vitro* passage of a replication-feeble HIV-1 clone carrying a CCR5-tropic *env* from a clinical isolate CRF08_BC. Sequencing the four adapted clones tested revealed multiple Env mutations, including the recurrent substitution at the gp120 residue E433 within the β20-β21 hairpin in the C4 domain. By using chimeric proviral and virus mutant clones, we found that viral infectivity enhancement required specific combinations of Env mutations. In one adapted clone, a four–amino acid deletion in the V2 domain, E433K, and a two-nucleotide insertion in gp41 that results in C-terminal truncation of the cytoplasmic tail acted cooperatively: only the triple combination reproduced the high infectivity of the adapted virus. In another adapted clone, while substitutions D166N and N195K in the V2 domain were insufficient, adding the E433K mutation to the two significantly elevated viral infectivity. Across the point mutants, D166N decreased infectivity whereas N195K increased infectivity. E433K alone was largely silent but compensated the D166N defect and potentiated the N195K effect. Introducing a positive charge at the 433 site strongly enhanced viral infectivity: E433R increased infectivity by ~five-fold on its own and markedly enhanced infectivity with D166N+N195K, whereas E433G had little impact. Mutants bearing D166N+N195K and/or E433R displayed increased sensitivity to soluble CD4, consistent with enhanced CD4-triggered Env responsiveness. In contrast, a CCR5-antagonist time-of-addition entry assay showed similar progression to CCR5 engagement, suggesting improved entry yield rather than faster entry kinetics. Neutralization profiling suggested limited global trimer remodeling, with mutation-dependent changes in V1/V2- and V3-glycan–directed antibodies but little impact on CD4-binding-site antibodies. *In silico* gp120–CD4 analyses supported this view, implicating inter-protomer V2 contacts and a positive-charge effect at E433 at the CD4 interface. Taken all together, these results identify E433 in the β20-β21 hairpin in the C4 domain as an electrostatic tuning site that cooperates with V2 and gp41 changes to increase productive entry.

## 1 Introduction

HIV-1 entry is a process that begins with Env-receptor binding and progresses to viral-host membrane fusion (Checkley et al., 2011; Chen, 2019; Murphy and Saad, 2020; Bennett and Henderson, 2021; Zhao et al., 2022). HIV-1 Env forms a trimer consisting of heterodimers of receptor-binding gp120 and membrane-anchoring gp41. The binding to the receptor CD4 induces structural changes in Env-gp120 to allow Env to bind to the coreceptor CCR5/CXCR4 followed by the membrane fusion mediated by Env-gp41 (Checkley et al., 2011; Chen, 2019; Murphy and Saad, 2020; Bennett and Henderson, 2021; Zhao et al., 2022).

The engagement of HIV-1 Env with CD4 causes Env conformational changes from the prefusion, closed state to the final open state (Wang et al., 2016, 2018; Bennett et al., 2024). It has been reported that during the conformational changes, Env adopts several intermediate states of structure (Bennett et al., 2024; Ozorowski et al., 2017; Yang et al., 2022; Dam et al., 2023; Li et al., 2023). Based on its amino acid sequence, HIV-1 Env-gp120 is divided into variable (V1–V5) and constant (C1–C5) domains. In a prefusion, closed state, V1V2 loops form the Env trimer apex and hide V3 having the coreceptor binding site underneath V1V2 loops. Structural features of a CD4-bound open Env have been shown to be the rotation of the gp120 core to the outside, the displacement of V1V2 loops from the apex, and the exposure of the V3 loop (Wang et al., 2016, 2018; Bennett et al., 2024; Dam et al., 2023; Li et al., 2023; Julien et al., 2013; Lyumkis et al., 2013). During the transition from closed to open states, structural changes also occur in β20β21 strands within the C4 domain and in β2β3 strands located downstream and upstream of V1V2 loops, respectively (Wang et al., 2016, 2018; Bennett et al., 2024; Dam et al., 2023; Li et al., 2023). The binding of Env to CD4 induces the rearrangement of the V1V2 loop, V3 loop, and β20β21 strands. Although CD4 contact sites are distributed across gp120, prominent contacts occur near the β2 strand and within the β20-β21 hairpin (Kwong et al., 1998; Herschhorn et al., 2017; Pancera et al., 2017), implicating these elements in CD4-binding and in CD4-triggered conformational transitions that influence infectivity.

Beyond gp120′s receptor binding role, multiple gp41 elements, including heptad repeat 1 and 2 (HR1/HR2), the membrane-proximal external region (MPER), the transmembrane domain, and the cytoplasmic tail (CT), also modulate viral infectivity and fusion capacity (Keller et al., 2018; Lu et al., 2019; Murakami and Freed, 2000; da Silva et al., 2016). Crosstalk between gp120 and gp41 (e.g., gp120–HR1 coupling) can regulate CD4-induced conformational changes, and the gp41 CT can influence trimer conformation, indicating that gp120 and gp41 jointly shape CD4-triggered Env dynamics (Keller et al., 2018; Chen et al., 2015; Finzi et al., 2010). However, the interactions between gp120 and gp41 and among intra Env domains that determine Env-CD4 engagement and infectivity have not yet been fully delineated.

HIV-1 exhibits high mutational and adaptive capacity. To pinpoint sequence determinants that govern Env-gp120/gp41 domain coupling and CD4-triggered conformational dynamics, an experimental adaptation approach that selects for infectivity enhancing mutations offers a direct path to discovery. Analyses of adapted viruses have identified genomic sites/regions and proteins involved in phenotypic change. Numerous *in vitro* HIV-1 adaptation studies have been conducted using diverse viral strains and experimental settings that impose distinct selective pressures on entry and replication, such as prolonged passage in primary cells, adaptation to restrictive host-cell environments, or selection for altered receptor usage (Pugach et al., 2004; Nomaguchi et al., 2013a; Beauparlant et al., 2017; Doi et al., 2019). Because Env is highly mutable, many mutations that modulate Env function have been reported, and one of the most prominent phenotypic outcomes of *in vitro* adaptation is enhanced Env-CD4 interaction and/or CD4-triggered Env responsiveness (Pugach et al., 2004; Humes et al., 2012; Beauparlant et al., 2017; Doi et al., 2019).

Building on this observation, we sought to define how specific Env domains interact to modulate viral infectivity and Env function using our efficient experimental adaptation system (Nomaguchi et al., 2013a; Doi et al., 2019; Koma et al., 2021; Yokoyama et al., 2016). Using an HIV-1 clone with poor replicative capacity that encodes *env* from a clinical isolate, we found that adaptive mutations in the V2, C4, and gp41 regions act cooperatively, and importantly, a triad of these mutations synergistically enhances infectivity. We further show that the E433 (HXB2 position 429) residue within the β20-β21 strands of the C4 region modulates infectivity via electrostatic effects. Together, these findings provide insights into how cooperative epistasis among Env domains enhances HIV-1 infectivity.

## 2 Results

### *2.1 In vitro* adaptation and selection of growth-enhanced viral clones

*In vitro* adaptation is an effective approach to obtain viral clones with adaptive mutations. By utilizing a long-term cell culture infected with authentically replication-inefficient viruses, growth-enhanced adaptive mutants are readily obtained (Nomaguchi et al., 2013a; Doi et al., 2019; Koma et al., 2021; Yokoyama et al., 2016).

Through long-term *in vitro* adaptation using the virus (Y3) carrying a CCR5-tropic *env* gene from an HIV-1 clinical isolate (CRF08_BC) (Doi et al., 2017), we isolated viral clones with improved growth in cell culture (Supplementary Figures 1, 2). Genome sequencing of four adapted clones (Y3ad-2,−6,−7, and−8) revealed multiple mutations (Table 1). Notably, E433K mutation within the β20β21 region in Env-gp120-C4 was found in all adapted clones sequenced. We selected Y3ad-6 (highest growth) and Y3ad-2 (fewest mutation sites) for further analysis.

**Table 1:** Mutations found in growth-enhanced Y3ad clones.

Y3ad-2			Y3ad-6			Y3ad-7			Y3ad-8		
Regions	Nt mutations	AA mutations	Regions	Nt mutations	AA mutations	Regions	Nt mutations	AA mutations	Regions	Nt mutations	AA mutations
									Vpr	g254a	R85Q
Tat^a^	g185a	S62N	Tat^a^	g185a	S62N	Tat^a^	g185a	S62N	Tat^a^	g185a	S62N
Rev^a^	g46a	V16M	Rev^a^	g46a	V16M	Rev^a^	g46a	V16M	Rev^a^	g46a	V16M
			Env (V2)	481–492del	161STEE164del						
Env (V2)	g496a	D166N							Env (V2)	g496a	D166N
			Env (V2)	g507a	Q169caa						
						Env (V2)	g550a	E184K			
Env (V2)	c585a	N195K									
Env (C4)	g1297a	E433K	Env (C4)	g1297a	E433K	Env (C4)	g1297a	E433K	Env (C4)	g1297a	E433K
			Env (gp41)	2460 aa insertion	gp41 deletion	Env (gp41)	2460 aa insertion	gp41 deletion	Env (gp41)	2460 aa insertion	gp41 deletion
						Nef	1–3 del	Start codon missing			
									Nef	c22t	R8C
									Nef	t519c	H173cac
			Nef	620–627 del	N207T, C208S, and 8 amino acids addition						
LTR	c386t										
			LTR	c458t							
						LTR	g465a		LTR	g465a	
LTR	g469a										

Four Y3ad clones were generated using the total DNA prepared from fresh M1.3S cells that were inoculated with the culture supernatant on day 123 of the long-term culture (Supplementary Materials and Methods and Supplementary Figures 1, 2).

Nucleotide (Nt) and amino acid (AA) mutations in the regions are shown as indicated.

^a^A single mutation within the overlapping region of *tat*- and *rev*-coding sequences.

### 2.2 Multiple mutations found in Y3ad-6 Env are responsible for viral growth enhancement

Clone Y3ad-6, which exhibited the highest growth potential, carried multiple mutations across the genome, including in Tat/Rev, Env-gp120/-gp41, Nef, and the LTR (Table 1). To identify adaptive (growth-enhancing) mutations, we constructed a series of chimeric clones between the parental Y3 and the adapted Y3ad-6 using shared unique restriction enzyme sites (Figures 1, 2A). These chimeric clones carried different combinations of adaptive mutations in Tat/Rev, Env-gp120, Env-gp41, Nef, and the LTR (Table 1, Figures 2A, B).

**Figure 1:**
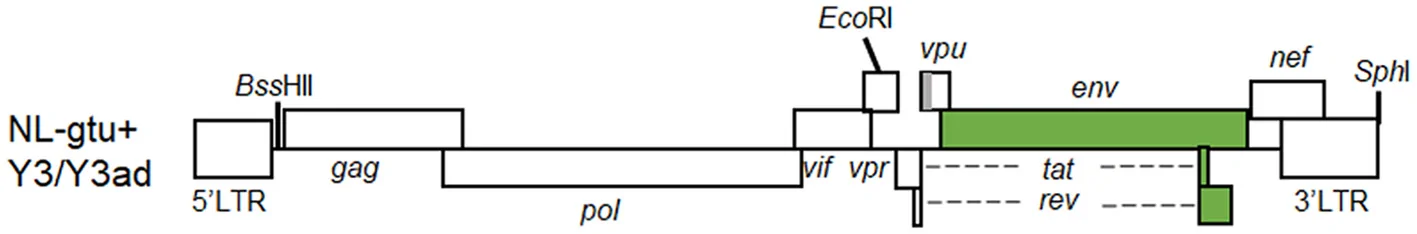
Genome organization of an HIV-1 clone used in this study. Genomes of NL-gtu+Y3/NL-gtu+Y3ad are schematically shown. White and gray areas represent the sequences from HIV-1(NL4-3) (Adachi et al., 1986) and SIVgsn166 (GenBank: AF468659), respectively. Green boxes show the *env* sequence from a clinical isolate (GenBank: LC816726). The unique restriction enzyme sites of the genomes used to generate HIV-1 clones are indicated.

**Figure 2:**
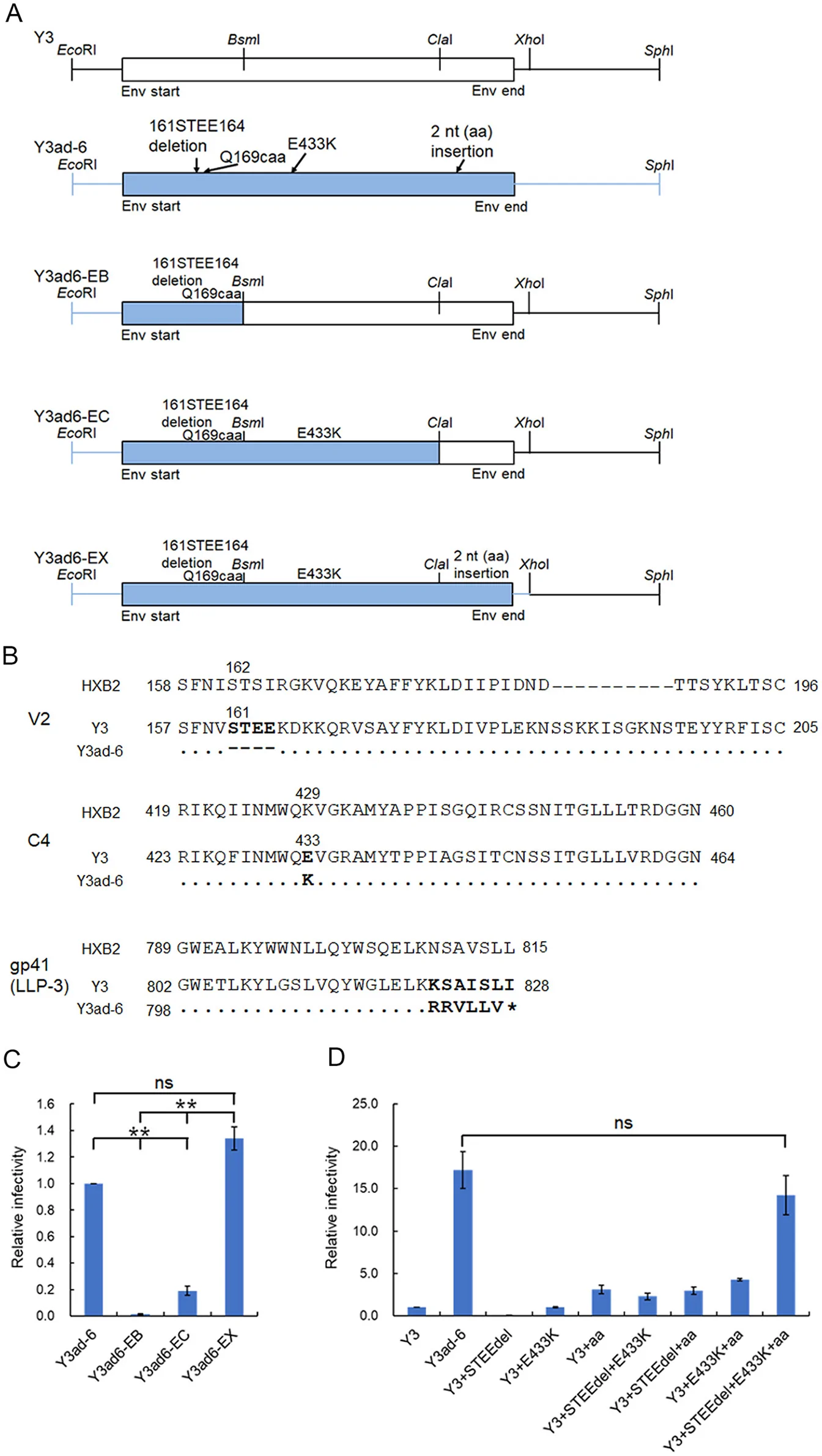
Identification of growth-enhancing mutations in Y3ad-6 clone. **(A)** Structures of chimeric viral clones. The regions from parental NL-gtu+Y3 (Y3) and NL-gtu+Y3ad-6 (Y3ad-6) clones are shown by white and blue boxes, respectively. The unique restriction sites used to generate chimeric virus clones are indicated. Mutations found in Y3ad-6 *env* are shown as indicated (also see Table 1). nt, nucleotide. **(B)** Env sequences of HXB2 and Y3. Mutations found in the V2, C4 and gp41 regions of Y3ad-6 Env are presented. Dots show the identical amino acid residues with those in the Y3 Env sequence. Dashes indicate the deletion of amino acid residues. Asterisk shows the presence of stop codon. Sequence alignments were generated by Genetyx ver. 14. Numbers indicate the corresponding amino acid positions in the HXB2 and Y3 Env sequences used throughout this study. LLP-3, lentivirus lytic peptide 3. **(C, D)** Effect of mutations on viral infectivity. Viruses were prepared from HEK293T cells transfected with the indicated clones. TZM-bl cells were infected with equal amounts of viruses. Luciferase activity in the TZM-bl cell lysates prepared on day 2 post-infection was measured and the infectivity of each virus sample relative to that for Y3ad-6 **(C)** and Y3 **(D)** was determined. Mean values ± standard errors (SE) from three independent experiments are shown. See Supplementary Tables 1, 2 for *p*-values by the Welch's *t*-test. **, *p* < 0.01; ns, not significant.

Three chimeric clones (Y3ad6-EB, -EC, and -EX), together with the adapted Y3ad-6 clone, were transfected into HEK293T cells to prepare input virus samples for inoculation into TZM-bl cells (Figure 2C). Although Y3ad6-EB, which carries Tat/Rev mutations and a four–amino acid deletion in the V2 domain of Env-gp120, exhibited extremely low infectivity, Y3ad6-EC modestly restored infectivity approximately 0.2 compared with the Y3ad-6. Notably, among the chimeric clones tested, only Y3ad6-EX, which contains all mutations except those in Nef and the LTR, exhibited infectivity comparable to that of Y3ad-6 (Figure 2C).

Consistent with the single-cycle infectivity assay, multi-cycle replication analyses showed that only Y3ad6-EX replicated at levels comparable to Y3ad-6 (Supplementary Figure 3). These results suggest that enhancement of virus infectivity requires a specific combination of adaptive mutations rather than individual changes. Because Y3ad6-EX exhibited comparable phenotypes in both multi-cycle replication and single-cycle infectivity assays (Figure 2C and Supplementary Figure 3), TZM-bl cells were used for evaluation of virus replication enhancement hereafter.

Although the Y3ad6-EX clone carries a mutation in Tat/Rev together with all adaptive mutations found in Env, the Y3ad6-EB clone, which harbors mutations in Tat/Rev and the Env-gp120-V2 region, did not exhibit enhanced viral infectivity (Figure 2C). Given the established roles of Tat and Rev in transactivation of HIV-1 transcription and nuclear export of viral mRNAs, respectively, these observations indicate that the Tat/Rev mutation is unlikely to contribute substantially to the enhancement of viral growth. Consistently, the finding that Y3ad6-EX displayed growth kinetics comparable to those of the adapted Y3ad-6 clone (Figure 2C and Supplementary Figure 3) suggests that neither the Nef nor the LTR regions harbor growth-enhancing adaptive mutations. Taken together, these results imply that multiple mutations in Env present in Y3ad6-EX are primarily responsible for the increased viral growth potential.

The Env of Y3ad-6 contains a four-amino acid deletion in the V2 domain (STEEdel), an E433K (HXB2 position 429) substitution in the C4 domain, and a two-nucleotide (aa) insertion in gp41 that introduces a frameshift, adding non-native amino acids and truncating the last 42 amino acids including LLP-1 (Table 1 and Figure 2B). To determine which of these mutations contribute to viral adaptation, we generated a series of proviral clones carrying individual, double, or triple combinations of the Env mutations found in Y3ad-6. The aa insertion in Env-gp41 alone increased viral infectivity relative to Y3, whereas the STEEdel mutation resulted in attenuation (Figure 2D). The E433K substitution by itself did not significantly affect viral infectivity. Among the double-mutant clones, inclusion of the E433K mutation consistently enhanced viral infectivity when combined with either STEEdel or the aa insertion; however, the level of enhancement did not reach that observed for the adapted Y3ad-6 clone (Figure 2D). In contrast, the triple-mutant clone (STEEdel + E433K + aa) exhibited viral infectivity comparable to that of Y3ad-6. These results demonstrate that a specific combination of three mutations in Env cooperatively contributes to the enhanced viral infectivity of the adapted clone.

### 2.3 Growth enhancement of Y3ad-2 results from combined effects of mutations in the Env-gp120-V2 and -C4 domains

To map adaptive mutations in Y3ad-2 similarly to Y3ad-6, we generated two chimeric clones (Y3ad2-EB and -EC) that contain various mutations in Tat/Rev, Env-gp120-V2 domain, Env-gp120-C4 domain, and LTR (Table 1 and Figures 3A, B). Virus samples were then prepared from HEK293T cells transfected with the proviral clones and inoculated into TZM-bl cells. As shown in Figure 3C, Y3ad2-EB, which contains V2 mutations D166N (HXB2 position 167) and N195K, showed around 3-fold lower infectivity relative to Y3ad-2. By contrast, Y3ad2-EC, which carries V2 (D166N and N195K) plus C4 (E433K), displayed infectivity comparable to Y3ad-2, indicating that combined V2 and C4 changes are required to sustain the enhanced infectivity (Figure 3C).

**Figure 3:**
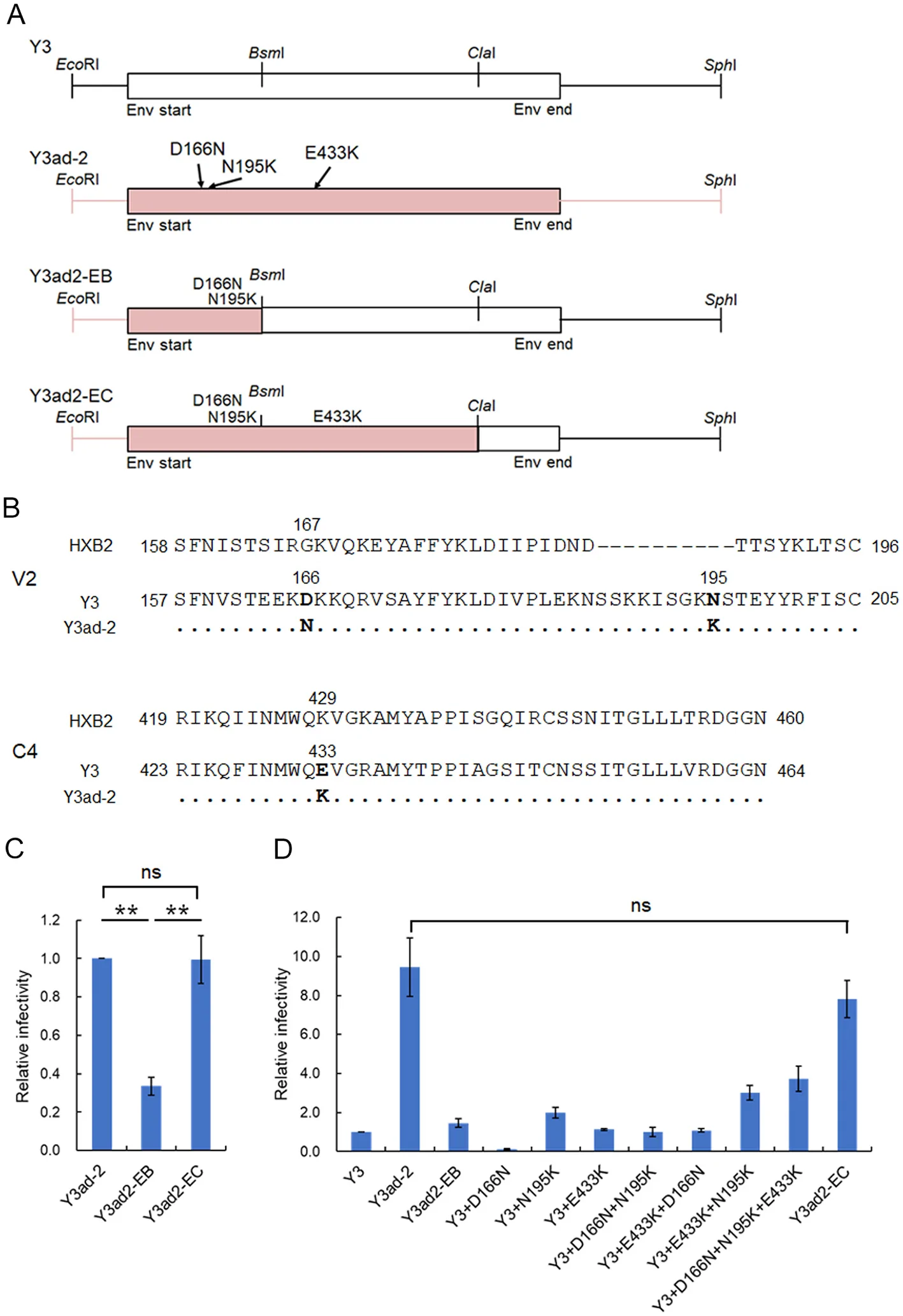
Identification of growth-enhancing mutations in Y3ad-2 clone. **(A)** Structures of chimeric viral clones. The regions from parental NL-gtu+Y3 (Y3) and NL-gtu-Y3ad-2 (Y3ad-2) clones are shown by white and pink boxes, respectively. The unique restriction sites used to generate chimeric virus clones are indicated. Mutations found in Y3ad-2 *env* are indicated (also see Table 1). **(B)** Env sequences of HXB2 and Y3. Mutations found in the V2 and C4 regions of Y3ad-2 Env are presented. Dots show the identical amino acid residues with those in the Y3 Env sequence. Sequence alignments were generated by Genetyx ver. 14. Numbers indicate the corresponding amino acid positions in the HXB2 and Y3 Env sequences used throughout this study. **(C, D)** Effect of mutations on viral infectivity. Infectivity of Y3, Y3ad-2, and Y3ad2-derived clones was analyzed as described in the legend to Figure 2. Virus infectivity was presented as the luciferase activity for each virus relative to that for Y3ad-2 **(C)** and Y3 **(D)**. Mean values ± SE from four independent experiments are shown. See Supplementary Tables 3, 4 for *p*-values by the Welch's *t*-test. **, *p* < 0.01; ns, not significant.

To dissect the contribution of individual Env mutations in the Y3 background, we constructed single, double, and triple mutants and quantified infectivity using TZM-bl assays (Figure 3D). D166N alone significantly reduced infectivity relative to Y3, whereas N195K alone induced an approximately 2-fold increase in infectivity. E433K alone had minimal effect. The D166N+N195K double mutant exhibited infectivity comparable to that of Y3, suggesting partial antagonism between the effects of the individual mutations. Notably, introduction of E433K into the D166N and D166N+N195K backgrounds restored infectivity to approximately 1.0-fold and increased it to approximately 4.0-fold relative to Y3, respectively, indicating that E433K compensates for the D166N-associated defect and enhances infectivity in combination with the V2 mutations. The N195K+E433K double mutant exhibited higher infectivity than N195K alone, and the infectivity of the triple mutant was consistently higher than that of N195K+E433K, although the difference was not statistically significant (Supplementary Table 4).

Among constructs that differ only by the presence or absence of the Tat/Rev change, clones carrying the Tat/Rev substitutions showed consistently higher mean infectivity than their Env-only counterparts (e.g., Y3ad-2 vs. Env triple mutant; Y3ad2-EB vs. V2 double mutant), although these differences did not reach statistical significance (Figure 3D and Supplementary Table 4). These observations raise the possibility that the Tat/Rev substitutions might make a minor contribution to specific infectivity in this background. Collectively, these data indicate that the infectivity enhancement in Y3ad-2 primarily arises from the combined effects of V2 (particularly N195K) and C4 E433K, with E433K mitigating the deleterious impact of D166N, and that Tat/Rev changes may provide a small additional benefit.

### 2.4 Substitution of E433 in the Env-gp120-C4 domain with positively charged residues (K or R) further enhances virus infectivity increased by adaptive mutations in the Env-gp120-V2 domain

Analysis of adaptive mutations in Y3ad-2 and Y3ad-6 revealed that the E433K substitution markedly elevated virus infectivity in specific combinations with other Env adaptive mutations. Residue E433 is located within the β20-β21 hairpin of the Env-gp120-C4 domain and directly contacts the CD4 receptor (Kwong et al., 1998; Herschhorn et al., 2017; Pancera et al., 2017). The residue at position 433 in Y3 Env (corresponding to position 429 in HXB2 numbering; Figure 4A) is relatively variable despite its location within the highly conserved β20-β21 strands. Indeed, non-E residues at this position have been reported in approximately 55% of 2,500 primary HIV-1 strains across all subtypes (Herschhorn et al., 2017).

**Figure 4:**
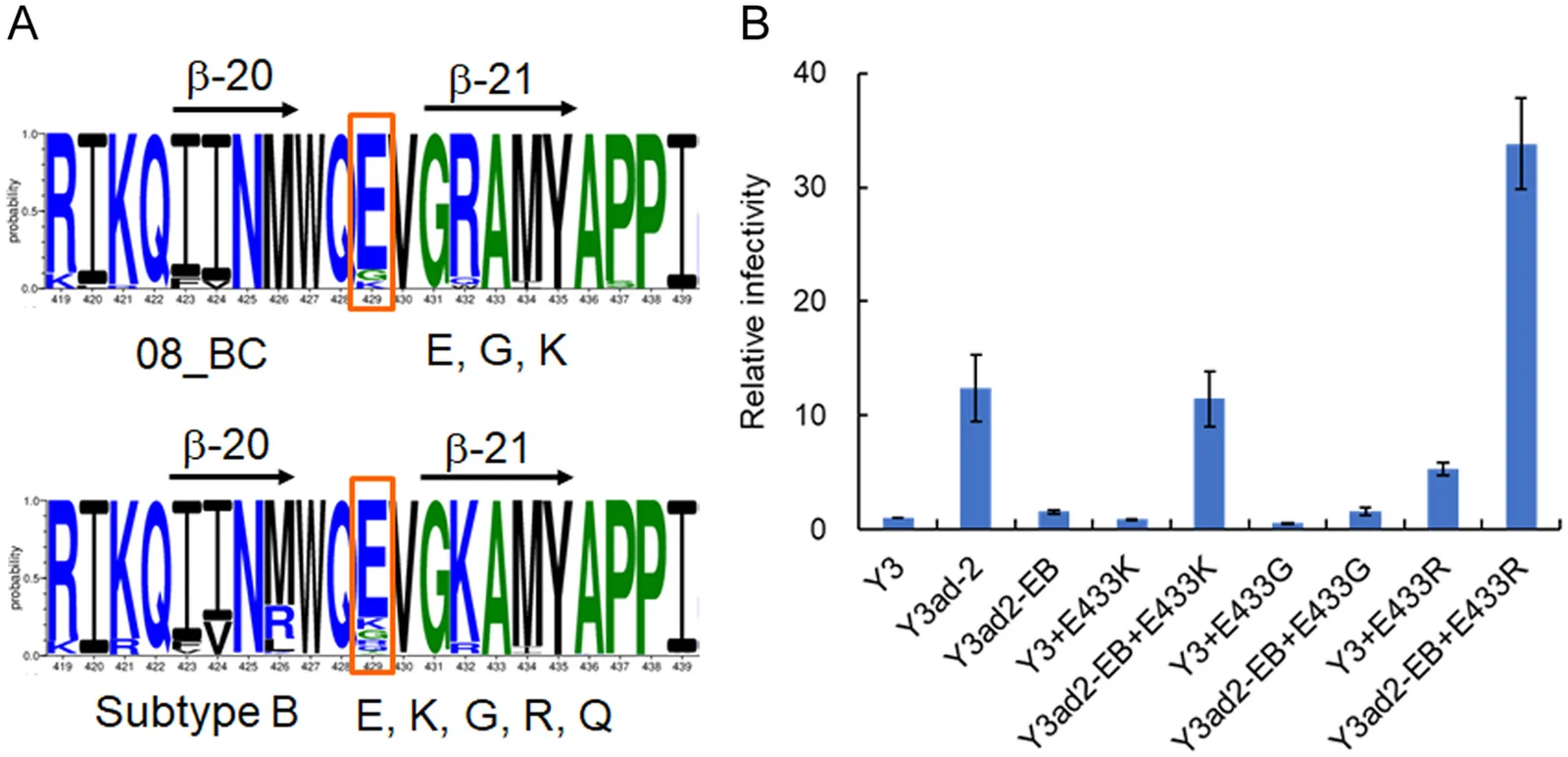
Effect of amino acid mutations at the E433 residue on virus infectivity. **(A)** Sequence analysis. The web logos of the sequences around the β20ß21 strands of subtype CRF08_BC (41 sequences) and B (2,334 sequences) were generated using the HIV-1 sequence database (https://www.hiv.lanl.gov/content/index). The HXB2 numbering of Env sequence is presented at the bottom of the web logos. The location of the β20-β21 strands is indicated. Orange boxes show the Y3-E433 site. **(B)** Virus infectivity. Infectivity of Y3, Y3ad-2, and Y3ad2-derived clones was analyzed as described in the legend to Figure 2. Mean values ± SE from four independent experiments are shown. See Supplementary Table 5 for the mean values ± SE and *p*-values by the Welch's *t*-test.

Analysis of Env-gp120-C4 sequences in the HIV-1 sequence database (https://www.hiv.lanl.gov/content/index) showed that the residue at position 433 in subtype CRF08_BC, to which Y3 belongs, exhibits variability, including K (identified here as an adaptive mutation) and G, in addition to the most frequent E (Figure 4A). Due to the limited number of available CRF08_BC sequences (n = 41), Env-gp120-C4 sequences from subtype B strains (n = 2,334) were further examined. Similar to CRF08_BC, multiple amino acid residues, including K, G, R, and Q, were observed at the position corresponding to Y3 Env residue 433 (Figure 4A).

To assess the functional impact of these substitutions, we generated Y3 and Y3ad2-EB (containing the V2 adaptive mutations D166N and N195K) variants carrying E433K, E433G, or E433R substitutions. Consistent with previous observations, the V2 adaptive mutations (D166N and N195K) alone, or E433K alone, had minimal effects on virus infectivity (Figure 4B). However, introduction of E433K into the clone carrying D166N and N195K increased virus infectivity to a level comparable to that of Y3ad-2. In contrast, the E433G substitution had little effect on infectivity, either alone or in combination with the V2 adaptive mutations (Figure 4B).

Notably, substitution of E433 with the positively charged residue R significantly enhanced virus infectivity even in the absence of other adaptive mutations, resulting in an approximately five-fold increase relative to parental Y3. Moreover, the E433R mutation synergistically enhanced the infectivity along with the D166N and N195K mutations (Figure 4B). Collectively, these results demonstrate that the amino acid property of the residue at position 433 of Env critically influences virus infectivity. Among naturally occurring variants, while E433G has little biological impact, E433K and E433R markedly enhance infectivity, particularly by potentiating the effects of adaptive mutations in the V2 domain. These findings suggest that electrostatic effects at the E433 site can play an important role in regulating HIV-1 infectivity.

### 2.5 Growth-enhancing mutations D166N and N195K in the Env-gp120-V2 and E433R in the Env-gp120-C4 can vary virus sensitivity to neutralization antibodies

Positively charged substitutions at E433 (K or R) within the β20-β21 hairpin of Env-gp120-C4 further enhanced virus infectivity in the presence of mutations in Env-gp120-V2. Adapted viruses obtained from *in vitro* adaptation experiments can exhibit altered sensitivity to soluble CD4 (sCD4) and neutralizing antibodies (NAbs) (Pugach et al., 2004; Beauparlant et al., 2017; Moore and Ho, 1995; Wrin et al., 1995; Clapham and McKnight, 2002). Because changes in neutralization sensitivity often reflect shifts in Env trimer conformation, we examined how the growth-enhancing mutations affect antibody sensitivity. We focused on D166N/N195K (V2) and E433R (C4), which clearly increased infectivity in TZM-bl cells (Figure 4B). We tested Y3ad2 derivatives carrying D166N+N195K (V2) or D166N+N195K+E433R (V2+C4) in a standard TZM-bl neutralization assay. The NAbs evaluated were PGT145 and PG16 (V1/V2 quaternary structure directed), VRC03 (CD4-binding site), and PGT128 (V3-glycan site) (Walker et al., 2009, 2011; Wu et al., 2010; Pejchal et al., 2011).

As shown in Figure 5, three clones tested (parental Y3 and Y3ad2 derivative clones) were resistant to neutralization by PGT145. Consistent with this result, it has been reported that a pseudovirus with the Env from an isolate (98CN006) of CRF_08BC subtype to which Y3 belongs displayed resistance against PGT145 antibody (Walker et al., 2011). While parental Y3 was relatively refractory to neutralization by PG16, the two mutant viruses (D166N+N195K and D166N+N195K+E433R) were equally quite sensitive to PG16 (Figure 5). The low sensitivity of Y3 to PG16 may be explained by the observation that a pseudovirus with the Env of an isolate (98CN006) is resistant against PG9, clonally related antibody of PG16 (Walker et al., 2011). These observations suggest that V2 mutations modestly improve presentation of the V1/V2 apex epitope recognized by PG16, whereas E433R has little additional effect on PG16 sensitivity. In line with previous results (Walker et al., 2011), parental Y3 virus was sensitive to neutralization by the CD4 binding site antibody VRC03 and by the V3-glycan antibody PGT-128 (Figure 5). While D166N/N195K and E433R mutations did not affect much the sensitivity to VRC03, the sensitivity to PGT-128 antibody was modestly decreased by D166N+N195K and substantially increased by E433R with the V2 mutations. In sum, D166N+N195K increased sensitivity to PG16 with minimal effects on PGT145, whereas the addition of E433R markedly increased PGT128 sensitivity, with little change for VRC03. These data indicate that growth-enhancing mutations in V2 (D166N/N195K) and C4 (E433R) alter Env antigenicity, likely through localized changes in epitope presentation at the V1/V2 apex and V3-glycan regions.

**Figure 5:**
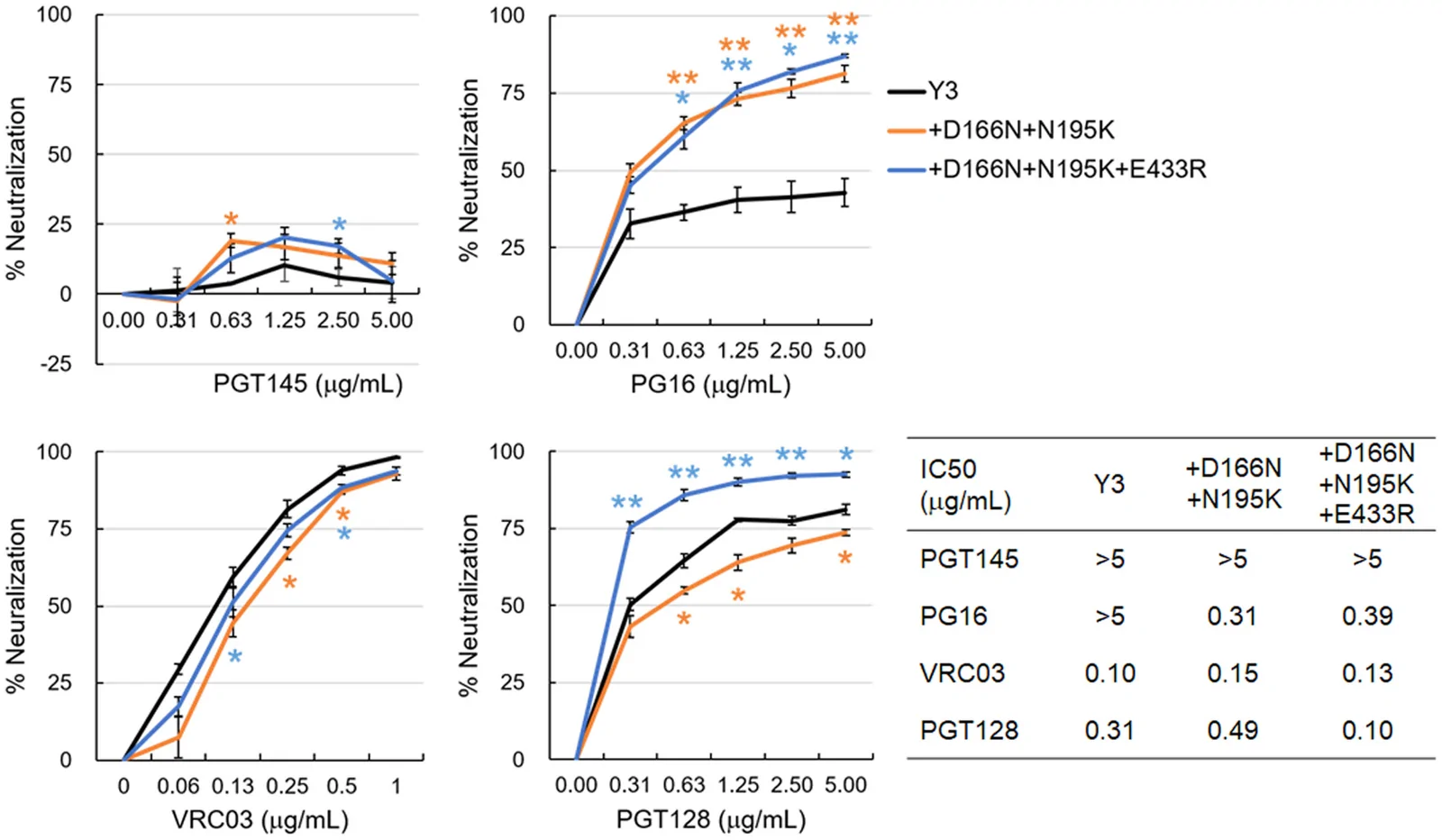
Effect of V2 (D166N and N195K) and C4 (E433R) mutations on neutralization sensitivity. Viruses were prepared from HEK293T transfected with the indicated NL-gtu+Y3 (Y3) or +Y3ad2-derived clones. Equal amounts of viruses were preincubated with appropriately diluted NAbs and inoculated into TZM-bl cells. On day 2 post-infection, the cells were lysed for luciferase assays. Percent neutralization of each virus was calculated from luciferase activity measured in the presence of the indicated concentration of NAb relative to that measured in the absence of NAb. Mean values ± SE from three independent experiments are shown. IC50 values were estimated from the neutralization curves. Significance relative to Y3 was determined by Welch's *t*-test (**p < 0.01; *p < 0.05; See Supplementary Table 6 for *p*-values). Orange and blue asterisks show the *p*-values for NL-gtu+Y3ad2 carrying D166N+N195K and D166N+N195K+E433R, respectively.

### 2.6 Enhancement of virus infectivity by growth-enhancing mutations D166N/N195K and E433R is attributable to increased Env-CD4 interaction

To investigate how the newly identified Env mutations (D166N/N195K and E433R) increase virus infectivity, we first examined virus entry kinetics. TZM-bl cells were attached with virus samples by spinoculation at 10 °C for 1 h, and then incubated at 37 °C for designated times (min) to enable viruses to enter into the cells. The CCR5 antagonist TAK-779 (Dragic et al., 2000) was added to the cells to stop the entry process. This assay quantifies the net progression through CD4-dependent early steps up to CCR5 engagement for each clone (Doi et al., 2019; Brandenberg et al., 2015). Because the CCR5 antagonist TAK 779 blocks CCR5 dependent entry and fusion, measurements at each addition time point represent events that had already engaged CCR5 and progressed to fusion. In addition to Y3 and Y3ad2 derivatives carrying D166N+N195K (V2) or D166N+N195K+E433R (V2+C4), a proviral clone carrying E433R alone was included. As can be seen in Figure 6A, all clones exhibited similar entry kinetics [area under the curve (AUC); 91.82 ± 11.01 for Y3, 81.52 ± 8.39 for D166N+N195K, 78.21 ± 12.91 for E433R, and 83.24 ± 7.17 for D166N+N195K+E433R]. No statistically significant differences were detected among variants in AUC-based comparisons, indicating that early entry progression as gauged at the CCR5 step is broadly comparable among these clones.

**Figure 6:**
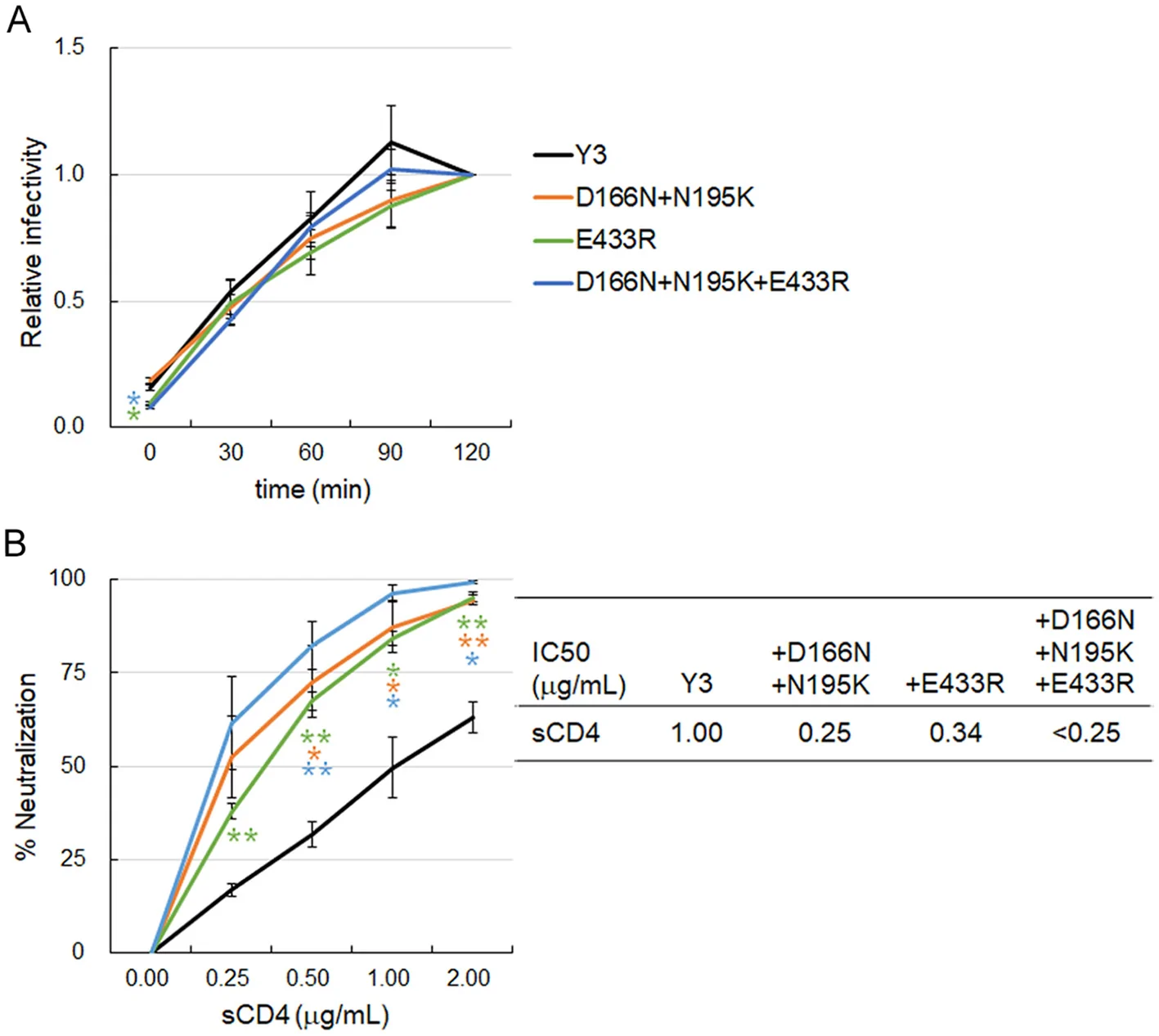
Effect of adaptive mutations on viral entry efficiency and sCD4 sensitivity. Viruses were prepared from HEK293T transfected with NL-gtu+Y3 (Y3) or +Y3ad2 derivative clones. **(A)** Entry kinetics assay. The assays were performed as described in the Materials and Methods. Infectivity was calculated as luciferase activity of each virus sample at the indicated time point relative to that at 120 min post-infection. **(B)** sCD4 sensitivity assays. Equal amounts of viruses were preincubated with appropriately diluted sCD4 and inoculated into TZM-bl cells. On day 2 post-infection, the cells were lysed for luciferase assays. Percent neutralization of each virus was calculated from luciferase activity measured in the presence of the indicated concentration of sCD4 relative to that measured in the absence of sCD4. Mean values ± SE from three independent experiments are shown. IC50 values were estimated from the neutralization curves. Significance relative to Y3 was determined by Welch's *t*-test (**p < 0.01; *p < 0.05; See Supplementary Table 7 for *p*-values). Green, orange and blue asterisks show the *p*-values for clones carrying E433R, D166N+N195K, and D166N+N195K+E433R, respectively.

It has been well documented that the increased sCD4 sensitivity of adapted viruses, which emerged during *in vitro* adaptation experiments, implies the enhanced Env-CD4 interaction (Pugach et al., 2004; Humes et al., 2012; Boyd et al., 2015; Del Prete et al., 2017; Doi et al., 2019; Koma et al., 2021). We carried out the sCD4 sensitivity assays using the four virus clones (Y3, D166N+N195K, E433R, and D166N+N195K+E433R). Similar to the neutralization assays described above, the preincubated mixture of virus samples and sCD4 of appropriate concentrations was inoculated into TZM-bl cells. As shown in Figure 6B, the three mutant viruses (D166N+N195K, E433R, and D166N+N195K+E433R) were neutralized at lower concentrations of sCD4 than parental Y3. The results on the three clones that showed increased sensitivity to sCD4 relative to parental Y3 implicate the enhancement of their Env-CD4 interactions.

Since our mutant clones tested (E433R, D166N+N195K, and D166N+N195K+E433R) showed an increase in Env-CD4 interaction, we analyzed the binding energy between their Env-gp120 monomers and CD4 *in silico* (Supplementary Materials and Methods and Supplementary Table 8). Although the values of binding energy slightly differed among Env-gp120s tested, no significant differences were observed (Supplementary Table 8). Together, these results support the conclusion that the infectivity enhancement conferred by D166N/N195K and E433R is associated with increased Env-CD4 interaction but not explained solely by a larger gp120-CD4 binding energy, whereas early entry kinetics *per se* remain similar when assessed through CD4-dependent early steps up to CCR5 engagement.

## 3 Discussion

Using our HIV-1 *in vitro* adaptation system, we identified strong epistatic cooperation among Env mutations in V2, C4 (β20-β21), and gp41 that robustly enhances viral infectivity. Individual changes were generally insufficient, whereas specific combinations produced pronounced gains, indicating that productive entry can be tuned by coordinated coupling among Env domains. In this network, residue E433 within the β20-β21 hairpin of Env-gp120-C4 is context dependent: E433K alone is largely silent, but it potentiates V2 and/or gp41 adaptive changes in both Y3ad-2 and Y3ad-6 backgrounds, highlighting E433 as a modulator of cooperative Env function.

A distinctive feature of the Y3ad-6 clone is a 2-nucleotide insertion in gp41 that truncates the cytoplasmic tail, including LLP-1. Consistent with prior work showing that gp41-CT alterations can influence Env incorporation and trimer conformation (Checkley et al., 2011; Chen, 2019; Murphy and Saad, 2020; Chen et al., 2015), this insertion modestly increased Env incorporation (Supplementary Figure 4). Importantly, this gp41 change participates in strong inter-domain epistasis: the V2 four-amino acid deletion (STEEdel) severely impaired infectivity, but either E433K or the gp41 insertion overcame this defect, and only the triple combination (STEEdel + E433K + gp41 insertion) reproduced the high infectivity of the adapted virus. These results underscore coordinated functional coupling among V2, C4 (including E433), and gp41, the mechanistic basis of which remains to be fully defined.

For the Y3ad-2-associated point mutations, systematic mutagenesis in the Y3 background revealed that D166N decreased infectivity, N195K increased infectivity, and E433K alone had minimal effect. Notably, adding E433K rescued the D166N defect and markedly increased infectivity in combination with the V2 mutations, and introducing a positive charge at 433 more broadly enhanced infectivity: E433R increased infectivity on its own and strongly potentiated infectivity with D166N+N195K, whereas E433G had little impact. Because E433 directly contacts CD4 (Kwong et al., 1998; Herschhorn et al., 2017; Pancera et al., 2017), these results support a model in which the side-chain property at position 433 acts as an electrostatic tuning element at the CD4 interface, whose functional consequences depend on compatible changes elsewhere in Env. Consistent with an increase in CD4-triggered Env responsiveness (Pugach et al., 2004; Humes et al., 2012; Beauparlant et al., 2017; Doi et al., 2019), mutants bearing D166N+N195K and/or E433R displayed increased sensitivity to sCD4. In contrast, TAK 779 time of addition profiles were broadly similar among variants, arguing against faster CCR5 engagement and instead suggesting improved entry yield under the conditions tested.

Neutralization patterns further argue against gross trimer remodeling. PGT145 (V1/V2 apex quaternary) (Walker et al., 2011; Lee et al., 2017) resistance remained essentially unchanged among the parental and mutant viruses. Although the parental Y3 Env was resistant to PGT145, PG16, a quaternary-structure-dependent broadly neutralizing antibody recognizing the V1/V2 apex epitope (Walker et al., 2009; Pancera et al., 2013), detected mutation-dependent differences in neutralization sensitivity. While the V2 mutations (D166N+N195K) increased sensitivity to PG16, addition of E433R had little further effect. Because D166 and N195 are located within the V1/V2 apex region, these mutations are likely to alter the local epitope environment recognized by PG16. In particular, the N195K substitution may abolish a potential N-linked glycosylation site and thereby modify glycan presentation around the apex, contributing to the enhanced PG16 sensitivity. In contrast, sensitivity to VRC03, which targets the CD4-binding site (Wu et al., 2010, 2011), remained largely unchanged despite the location of E433 within the β20-β21 region that directly contacts CD4. This observation suggests that the overall architecture and antigenic presentation of the CD4-binding site are largely preserved. Likewise, the absence of mutation-dependent changes in PGT145 sensitivity argues against major reorganization of the trimer apex. By contrast, PGT128 (V3-glycan) (Walker et al., 2011; Pejchal et al., 2011) sensitivity changed substantially in a mutation-dependent manner, decreasing with D166N+N195K and increasing upon addition of E433R. Because E433 is not part of the known PGT128 epitope, these effects are unlikely to arise from direct alteration of antibody-contact residues. Rather, they suggest that structural perturbations originating from the β20-β21 regulatory region can influence the V3-glycan epitope environment through long-range conformational communication within Env. Taken together, these neutralization profiles support localized alterations in epitope presentation around the V1/V2 apex and V3-glycan regions rather than global reorganization of the Env trimer.

These virological phenotypes are consistent with our *in silico* gp120-CD4 complex analyses, which provide a framework for the observed cooperative behavior. In the Y3 Env trimer model, the negatively charged D166 residue interacts with the positively charged K168 residue of a neighboring protomer (Figures 7 and 8). This suggests inter-protomer contact via electrostatic interactions, which may stabilize V2 at the trimer apex and influence conformational transitions (O'Rourke et al., 2012; Katte et al., 2025; Salzwedel and Berger, 2000). Disruption of this contact by D166N may be detrimental on its own, whereas N195K could retune the local V2 environment, potentially aided by loss of the N195 glycan, to favor an entry-competent configuration. Structural comparison of gp120-CD4 complexes and corresponding electrostatic surface representations (Figure 8) revealed localized changes around the V1/V2 region and the CD4-binding interface. Several CD4 residues, including K22, L44, K46, and R59, have previously been implicated in gp120 recognition (Kwong et al., 1998; Liu et al., 2017). Consistent with these previous findings, our structural models predicted that K22, S23, Q40, L44, K46, R59, E87, and D88 are positioned near the molecular surface altered by E433K/R. These residues may contribute to gp120-CD4 recognition through a combination of electrostatic and hydrogen-bond interactions at the CD4-binding interface. In agreement with this interpretation, both E433K and E433R potentiated infectivity in combination with the V2 changes, with E433R producing the strongest effects in our assays. In this regard, the stronger phenotype of E433R relative to E433K may reflect greater capacity of Arg than Lys to stabilize local electrostatic and hydrogen-bond networks at the CD4-contacting surface (Sokalingam et al., 2012), although the precise structural basis remains to be determined. Loss of the N195 glycan is predicted to remodel the local V2 environment and facilitate structural rearrangements around the V1/V2 apex, whereas E433R preserves a configuration that appears more compatible with CD4 engagement. Together, these observations support a model in which D166N/N195K and E433K/R collectively increase the probability of productive, CD4-triggered entry transitions rather than simply accelerating early entry steps. This model is consistent with previous reports suggesting functional coupling between the V1/V2 and C4 regions of Env (Freed and Martin, 1994; Zolla-Pazner et al., 2015; Powell et al., 2017). Indeed, in JR-FL Env, a β20-β21 mutation (I423V) is largely silent alone but amplifies phenotypes conferred by a V2 mutation (L193A), highlighting conditional β20-β21 behavior (Herschhorn et al., 2017).

**Figure 7:**
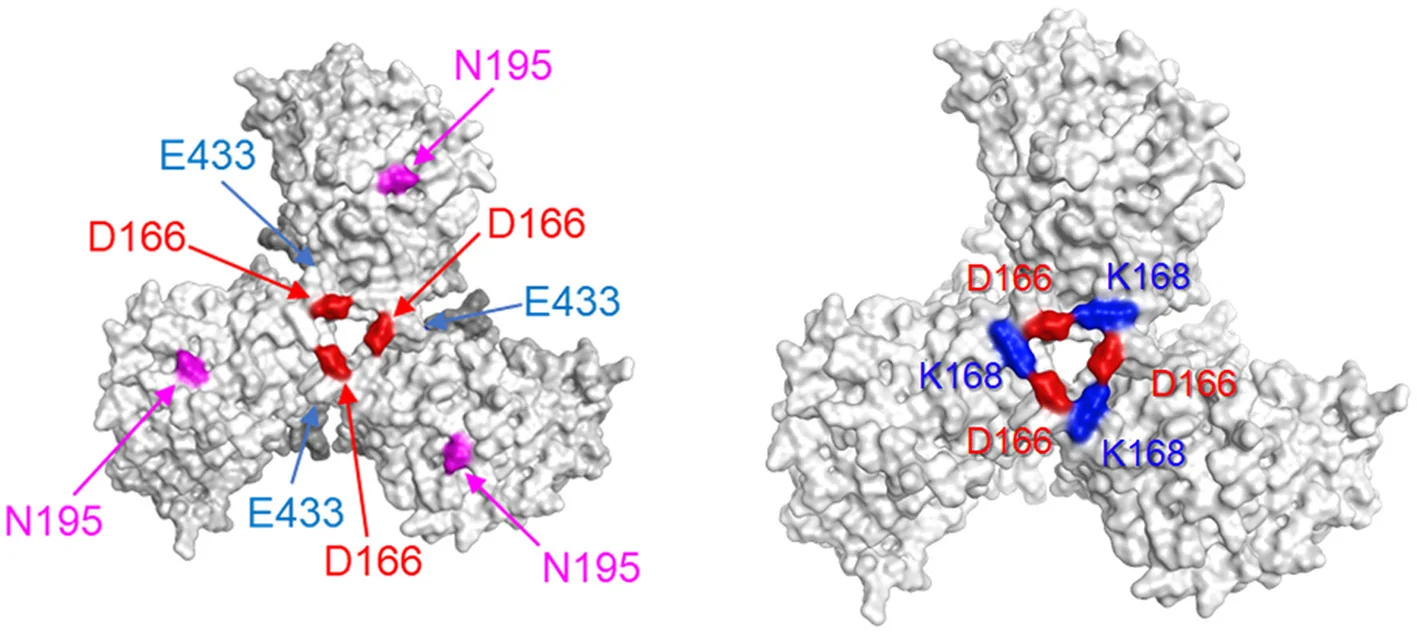
The location of adaptive mutation site. The surface representation of the gp120 trimer is illustrated in light gray. Left; Adaptive mutation sites found in Y3ad-2 (D166, N195, and E433) are indicated. Right; the surface of residues D166 (red) and K168 (blue) is highlighted to illustrate their spatial arrangement within the V1/V2 loop region. These residues are positioned at the trimer apex and form a cluster that may influence inter-subunit interactions and structural stability of the envelope glycoprotein.

**Figure 8:**
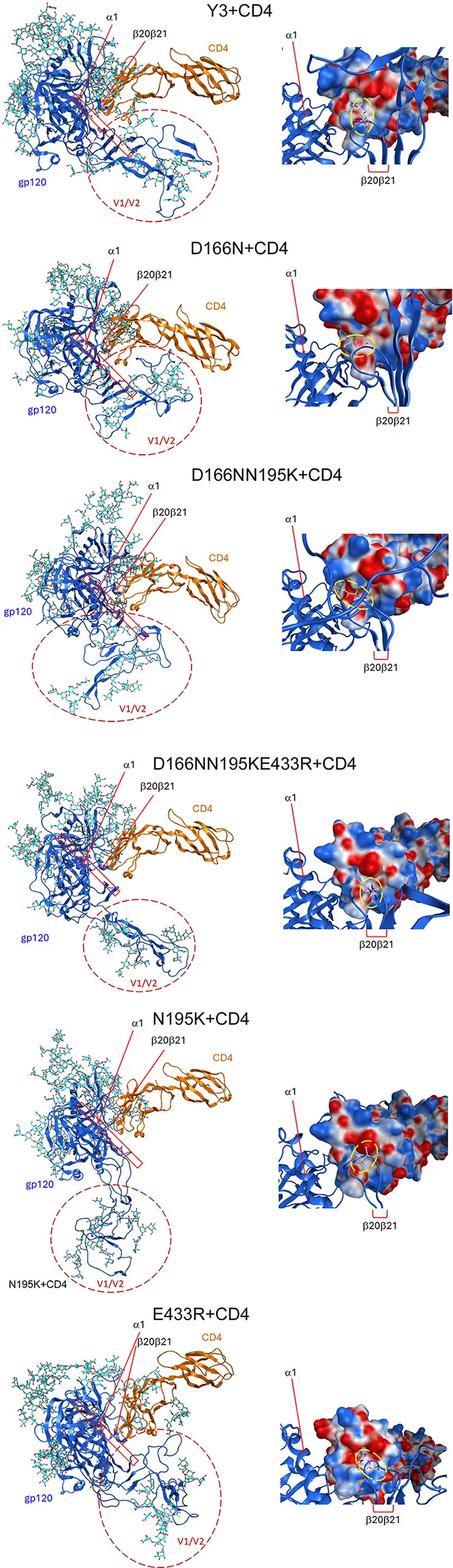
A molecular dynamics simulation analysis of HIV-1 gp120-CD4 complexes. Structural models of gp120-CD4 complexes at 100 ns (left) and corresponding close-up views of gp120 around residue 433 (right). Left: HIV-1 gp120 is shown in blue and CD4 in orange. The V1/V2 loop region is indicated by a red dashed circle. N-linked glycans are shown as stick representations (cyan). Right: Views are from the gp120 regions indicated by red boxes toward the gp120 surface surrounding residue 433. E433 or E433R is shown as a stick representation (white) and highlighted by a yellow dashed circle. Molecular surfaces are colored according to electrostatic potential, with red indicating negative charge and blue indicating positive charge. For easy comparison, the α1 helix and β20-β21 strands are indicated.

Analysis of the HIV sequence database (subtypes B and CRF08_BC) did not identify a specific V2 polymorphism strongly associated with positively charged residues at HXB2 position 429 (Supplementary Figures 5, 6). Together with our experimental findings, this observation suggests that E433K/R is not functionally linked to a single conserved V2 mutation. Rather, the β20-β21 residue E433 appears to function as a regulatory site that can cooperate with distinct Env changes in different genetic backgrounds. In addition, temporal analysis of HIV-1 subtype B sequences in the HIV Sequence Database did not reveal consistent enrichment of the adaptive mutations (D166N and E433K/R) identified in this study. Although the frequency of Arg (R) at HXB2 position 429 increased modestly between 2009 and 2022, no comparable increase was observed for Asn (N) at HXB2 position 167 (Supplementary Table 9). These observations suggest that, although the mutations identified here enhance infectivity in our experimental system, their prevalence in circulating viruses is likely influenced by additional selective pressures, including immune escape, Env stability, and transmission fitness.

Extending this concept beyond the Y3 isolate, we previously showed during adaptation of NL4-3 that E427K (corresponding to Y3 E433K) synergized with adaptive substitutions in the V1 and C2 regions to enhance viral replication (Doi et al., 2019). Taken together, these findings support a broader role of the β20-β21 regulatory region as an allosteric tuning site that modulates HIV-1 infectivity through cooperation with different Env mutations in distinct sequence contexts, enabling HIV-1 to rebalance CD4-triggered transitions and entry competence through cooperative epistasis. The identification of an allosteric regulatory site within the β20-β21 region may provide useful insights into mechanisms governing Env conformational control.

## 4 Materials and methods

### 4.1 Plasmid DNAs

A proviral clone gtu+Y3, which carries a CCR5-tropic *env* sequence from a clinical isolate (CRF08_BC subtype), has been described previously (GenBank: LC816726) (the same clone as gtu+CI3 of Doi et al., 2017). Adapted proviral Y3ad clones were generated by introducing the fragment from the *Eco*RI site in the *vpr* region to the *Sph*I site located at the 3′ end of viral genome into the corresponding site of the gtu+Y3 clone as described previously (Nomaguchi et al., 2013b). The generation of chimeric viruses between gtu+Y3/Y3ad-6 and those between gtu+Y3/Y3ad-2 clones were carried out by exchanging the DNA fragments using unique restriction enzyme sites on their genomes as shown in Figures 2, 3. NL-gtu+Y3/NL-gtu+Y3ad-2 and−6 clones were constructed by replacing the *Bss*HII-*Eco*RI fragment of gtu+Y3 and Y3ad clones, respectively (Figure 1), with the corresponding fragment of HIV-1(NL4-3) clone (Adachi et al., 1986). The introduction of mutations into the *env* genes was done by PCR-based site-directed mutagenesis. Mutant clones at the E433 residue in Env-gp120-C4 domain were generated by PCR-based site-directed mutagenesis using NL-gtu-Y3ad2-EB clone as a template.

### 4.2 Cells

A human embryonic kidney cell line HEK293T (Lebkowski et al., 1985) and a reporter cell line TZM-bl carrying a luciferase gene driven by viral LTR (Platt et al., 1998, 2009) were cultured in minimal essential medium (MEM) supplemented with 10% heat-inactivated fetal bovine serum.

### 4.3 Virus preparation and measurement of virus amount

Virus stocks were prepared from HEK293T cells transfected with proviral clones by the calcium phosphate co-precipitation method (Adachi et al., 1986; Kamada et al., 2006). Briefly, cells (6–7 x 10^5^) were seeded on a 6-well plate and the next day, appropriate proviral clones (2.5 μg) were transfected with ProFection Mammalian Transfection System (Promega) into the cells. On day 2 post-transfection, the culture supernatants were collected and stored in −80 °C until use. Amounts of viruses were determined by Gag-p24 ELISA (ZeptoMetrix Corporation).

### 4.4 Virus infectivity, neutralization, and entry kinetics assays

Virus infectivity, neutralization, and entry kinetics assays were performed similarly as previously described (Doi et al., 2019; Brandenberg et al., 2015). Briefly, TZM-bl cells (4.0 x 10^3^) were seeded on a 96-well plate. On the next day, equal amounts of virus stocks were inoculated into the TZM-bl cells. For neutralization assays, virus stocks were pretreated with sCD4 (catalog no. CYT-304; ProSpec-Tany Technogene Ltd.) or various anti-HIV-1 gp120 antibodies for 1 hr at 37 °C before inoculation. Following preincubation of virus samples and each sCD4 or NAb appropriately diluted, the mixture was inoculated into TZM-bl cells for virus infectivity assay. All anti-HIV-1 gp120 antibodies were obtained from NIH AIDS Reagent Program: PGT145 (catalog no. 12703), PG16 (catalog no. 12150), VRC03 (catalog no. 12032), and PGT128 (catalog no. 13352). On day 2 post-infection, cells were lysed for luciferase assays according to the manufacturer's instructions (Promega). Percent neutralization was calculated as 100 × [1 – (luciferase activity in the presence of NAb or sCD4 / luciferase activity in the absence of NAb or sCD4)]. IC50 values were estimated from dose–response curves using an online IC50 calculator (AAT Bioquest, https://www.aatbio.com/tools/ic50-calculator). For entry kinetics assays, virus-containing medium was spin-infected into TZM-bl cells at 10 °C for 1hr and replaced with the pre-warmed fresh culture medium. At appropriate time points (0 to 120 min post-spin-infection), a CCR5 antagonist TAK-779 (at a final concentration of 1 μM), was added to the culture media to stop viral entry. On day 2 post-infection, cells were lysed for luciferase assays according to the manufacturer's instructions (Promega).

### 4.5 Sequence analysis

Sequences of HIV-1 Env-gp120-C4 region were obtained from the HIV-1 sequence database (https://www.hiv.lanl.gov/content/index) on October 2, 2022. The web logos were made using the database web tool (https://www.hiv.lanl.gov/content/sequence/ANALYZEALIGN/analyze_align.html).

### 4.6 HIV-1 gp120-CD4 complexes modeling

The gp120-CD4 complex models were generated by means of a molecular modeling approach, employing the MOE 2022.02 software package (Chemical Computing Group Inc., Montreal, Quebec, Canada), as previously delineated (Doi et al., 2019; Yokoyama et al., 2016). The cryo-EM structure of the HIV-1 CD4-gp120 complex at 3.90 Å resolution [PDB ID: 6MEO, chains G and A (Shaik et al., 2019)] was utilized as the modeling template. The gp120 region of the complex was modeled using the sequence of the HIV-1 Y3 strain or its mutants (D166N, N195K, E433R, D166N+N195K, or D166N+N195K+E433R).

### 4.7 Molecular dynamics simulations

Molecular dynamics (MD) simulations were performed using the pmemd.cuda.MPI module from the Amber 24 program package (University of California, San Francisco), employing the Amber ff14SB (Maier et al., 2015) and GLYCAM_06j-1 (Kirschner et al., 2008) force fields. The TIP3P water model (Jorgensen et al., 1983) and a 0.15 M NaCl salt concentration were established around the gp120-CD4 complex. The bond lengths involving hydrogen were constrained using the SHAKE algorithm (Ryckaert et al., 1977), and the time step for all MD simulations was set to 2 fs. In this study, a nonbonded cutoff of 10 Å was employed. Heating calculations were performed for 20 ps until 310 K using the NVT ensemble. Subsequently, MD simulations were executed employing the NPT ensemble at 1 bar and 310 K for a duration of 100 ns.

## Data Availability

The original contributions presented in the study are included in the article/Supplementary material, further inquiries can be directed to the corresponding authors.
