# Supplementary material for: Natural variations at the E433 site in Env-gp120 β20-β21 cooperate with V2 and gp41 mutations to modulate HIV-1 infectivity
Source: Front Microbiol. 2026 Sep 15;17:1877899. doi: 10.3389/fmicb.2026.1877899 (PMC13619938; doi:10.3389/fmicb.2026.1877899)
Supplement: Supplementary file 1 [file Table_1.docx]

Supplementary Material

# 1 Supplementary Materials and Methods

**Cells**

A rhesus macaque lymphocyte cell line M1.3S (CD4+CCR5+CXCR4+) was maintained in RPMI1640 medium containing 10% heat-inactivated fetal bovine serum and 50 units/mL of recombinant human interleukin-2 (IL-2) (Bio-Rad Laboratories Inc.) (Doi et al., 2011).

**Construction of adapted viral clones**

The culture supernatant on day 123 was inoculated into fresh M1.3S cells and then total DNA was prepared from the cells (Supplementary Fig. 1). In order to identify the sites that impact Env functions, the region from the *vpr* gene to the 3’ end of the genome was amplified by PCR. Adapted virus clones were constructed by introducing the fragment from the *Eco*RI site in *vpr* to the *Sph*I site at 3’ end of the genomes into the parental Y3 clone (Y3ad) as described previously (Nomaguchi et al., 2013).

**Virus preparation and measurement of virus amount**

Virus stocks were prepared from HEK293T cells transfected with proviral clones by the calcium phosphate co-precipitation method (Adachi et al., 1986; Kamada et al., 2006). Amounts of viruses were determined by RT assay to measure virion-associated RT activity (Nomaguchi et al., 2013; Willey et al., 1988), by real-time RT-PCR to assess viral RNA copy numbers in virions (Koma et al., 2023), or by Gag-p24 ELISA (ZeptoMetrix Corporation).

**Multi-cycle replication**

Virus stocks were inoculated into M1.3S cells and the culture supernatants were collected every 3 days. Virus replication was monitored by RT activity or viral RNA copy numbers in the culture supernatants. In the long-term culture, fresh M1.3S cells were occasionally added to the culture to rescue persistent viruses.

**Calculation of binding free energy**

The binding free energies of CD4 to gp120 in solution were calculated from all-atom MD trajectories of gp120-CD4 complexes from the final 5 ns (100 frames) following 100 ns of MD simulations. The calculation was performed computationally using the MMPBSA.py tool (Miller et al., 2012) in the AmberTools24 (University of California, San Francisco). The free energy of binding in solution was calculated by the MM/PBSA and MM/GBSA methods, with consideration for solvent-solvent interactions, incorporating the free energy of binding in a vacuum (Miller et al., 2012).

**Sequence analysis of HIV-1 strains**

Filtered and super-filtered Env amino acid sequence alignments of HIV-1 subtype B and CRF08_BC isolates were obtained from the HIV Sequence Database (https://www.hiv.lanl.gov/content/index) on July 7, 2026. Sequence datasets were filtered and reformatted according to the amino acid present at position 433 (Y3 numbering) using AliView v1.31 (Larsson, 2014). The resulting sequence sets were subsequently realigned using Clustal Omega (https://www.ebi.ac.uk/jdispatcher/msa/clustalo) (Madeira et al., 2024).

Amino acid frequencies were analyzed using the HIV Sequence Database AnalyzeAlign tool (https://www.hiv.lanl.gov/content/sequence/ANALYZEALIGN/analyze_align.html). Sequence logos (Crooks et al., 2004) were generated for comparative analysis of subtype B and CRF08_BC sequences carrying different amino acids at position 433.

# 2 Supplementary Figures and Tables

**Supplementary Figure 1.** An adaptation experiment using an HIV-1 clone and M1.3S cells. (A) Schema of adaptation experiment. (B) The virus sample (2.3 x 10^7^ RT units) prepared from HEK293T cells transfected with the gtu+Y3 clone was inoculated into a lymphocyte cell line M1.3S (2.0 x 10^6^ cells). The infected cell culture was maintained by occasionally adding fresh M1.3S cells on the day shown by arrows with days post-infection. A black circle indicates culture supernatants on day 123 post-infection collected for generating adapted viral clones. (C) Viruses (2.5 x 10^6^ RT units) prepared from transfected HEK293T cells (open circle) and those in the supernatant collected from the long-term infected culture (closed circle) were inoculated into M1.3S cells. Culture supernatants were periodically collected, and viral replication was monitored by the RT activity of the supernatants.

**Supplementary Figure 2.** Growth kinetics of parental and adapted viral clones in M1.3S cells. The culture supernatant on day 123 was inoculated into fresh M1.3S cells (Supplementary Fig. 1) and then Y3ad clones were generated using total DNA prepared from the cells as described in the Supplementary Materials and Methods. Viruses were prepared from HEK293T cells transfected with the indicated proviral clones. Equal amounts of virus samples (10^6^ RT units) were inoculated into M1.3S (2.0 x 10^5^ cells). RT activity in the culture supernatants collected every 3 days post-infection was measured to monitor virus replication. The left panel shows the replication abilities of multiple adapted clones, whereas the right panel shows selected adapted clones examined in follow-up experiments.

**Supplementary Figure 3.** Effect of mutations on viral replication potential and infectivity. Viruses were prepared from HEK293T cells transfected with the indicated clones. M1.3S cells were infected with equal amounts of viruses (200 ng of Gag-p24). Virus replication potential was assessed by measuring viral RNA copy numbers in the culture supernatants collected every 3 days post-infection. The representative data from the two independent experiments are presented.

|  | Mean | SE | *p*-value |
| --- | --- | --- | --- |
| Y3 | 1.00 | 0 |  |
| Y3+aa | 1.50 | 0.18 | 0.0351 |

**Supplementary Figure 4.** Effect of gp41 mutations on Env incorporation into virions. Viruses were produced in HeLa and Hep2 cells transfected with the indicated proviral clones. Virions were pelleted by ultracentrifugation of culture supernatants and analyzed by Western blotting using anti-gp120 and anti-Gag-p24 antibodies. Band intensities were quantified using ImageJ, and Env incorporation was assessed as the gp120/p24 ratio. Values are shown as the mean of Y3+aa relative to Y3 (n = 6 total; three independent experiments in HeLa and three independent experiments in Hep2). The *p*-value was determined by the Welch's *t*-test. Representative blots from independent experiments are shown at the top.

E433K

E433

**Supplementary Figure 5.** Sequence logos of the V2 region from HIV-1 CRF08_BC viruses carrying E433 (left) or E433K (right). Sequence logos were generated as described in the Supplementary Materials and Methods. Amino acid sequences corresponding to HXB2 positions 158-196 were extracted from LANL HIV database sequences and aligned prior to logo generation. A total of 61 E433-containing and 5 E433K-containing CRF08_BC sequences were analyzed.

E433K/R

E433

**Supplementary Figure 6.** Sequence logos of the V2 region from HIV-1 subtype B viruses carrying E433 (left) or E433K/R (right). Sequence logos were generated as described in the Supplementary Materials and Methods. Amino acid sequences corresponding to HXB2 positions 158-196 were extracted from LANL HIV database sequences and aligned prior to logo generation. A total of 2296 E433-containing and 363 E433K/R-containing subtype B sequences were analyzed.

**Supplementary Table 1. Mean values ± SE and *p*-values for Y3ad-6 and its chimera clones (Fig. 2C)**

|  | Mean | SE | vs Y3ad6 | vs EB | vs EC |
| --- | --- | --- | --- | --- | --- |
| Y3ad-6 | 1.00 | 0.00 |  |  |  |
| Y3ad6-EB | 0.01 | 0.00 | 0.0000 |  |  |
| Y3ad6-EC | 0.19 | 0.03 | 0.0019 | 0.0361 |  |
| Y3ad6-EX | 1.34 | 0.09 | 0.0625 | 0.0044 | 0.0024 |

**Supplementary Table 2. Mean values ± SE and *p*-values for Y3ad-6 and its Env mutant clones (Fig. 2D)**

|  | Mean | SE |  |  |  |  |  |  |
| --- | --- | --- | --- | --- | --- | --- | --- | --- |
| Y3 | 1.00 | 0.00 |  |  |  |  |  |  |
| Y3ad-6 | 17.19 | 2.16 |  |  |  |  |  |  |
| Y3+STEEdel | 0.10 | 0.03 |  |  |  |  |  |  |
| Y3+E433K | 1.03 | 0.06 |  |  |  |  |  |  |
| Y3+aa | 3.13 | 0.48 |  |  |  |  |  |  |
| Y3+STEEdel+E433K | 2.31 | 0.39 |  |  |  |  |  |  |
| Y3+STEEdel+aa | 3.00 | 0.43 |  |  |  |  |  |  |
| Y3+E433K+aa | 4.31 | 0.15 |  |  |  |  |  |  |
| Y3+STEEdel+E433K+aa | 14.20 | 2.30 |  |  |  |  |  |  |
|  | vs Y3 | vs Y3ad-6 | vs +STEEdel | vs +E433K | vs +aa | vs +STEEdel +E433K | vs +STEEdel +aa | vs +E433K+aa |
| Y3 |  |  |  |  |  |  |  |  |
| Y3ad-6 | 0.0173 |  |  |  |  |  |  |  |
| Y3+STEEdel | 0.0009 | 0.0155 |  |  |  |  |  |  |
| Y3+E433K | 0.6281 | 0.0173 | 0.0009 |  |  |  |  |  |
| Y3+aa | 0.0467 | 0.0188 | 0.0237 | 0.0463 |  |  |  |  |
| Y3+STEEdel+E433K | 0.0779 | 0.0178 | 0.0290 | 0.0784 | 0.2534 |  |  |  |
| Y3+STEEdel+aa | 0.0438 | 0.0190 | 0.0212 | 0.0430 | 0.8473 | 0.2993 |  |  |
| Y3+E433K+aa | 0.0020 | 0.0264 | 0.0009 | 0.0006 | 0.1229 | 0.0240 | 0.0816 |  |
| Y3+STEEdel+E433K+aa | 0.0291 | 0.3975 | 0.0257 | 0.0292 | 0.0360 | 0.0325 | 0.0359 | 0.0497 |

**Supplementary Table 3. Mean values ± SE and *p*-values for Y3ad-2 and its Env mutant clones (Fig. 3C)**

|  | Mean | SE | vs Y3ad-2 | vs EB |
| --- | --- | --- | --- | --- |
| Y3ad-2 | 1.00 | 0.00 |  |  |
| Y3ad2-EB | 0.34 | 0.05 | 0.0008 |  |
| Y3ad2-EC | 0.99 | 0.12 | 0.9701 | 0.0049 |

**Supplementary Table 4. Mean values ± SE and *p*-values forY3ad-2 and its Env mutant clones (Fig. 3D)**

|  | Mean | SE | vs Y3 | vs Y3ad-2 | vs Y3ad2-EB |  |  |
| --- | --- | --- | --- | --- | --- | --- | --- |
| Y3 | 1.00 | 0.00 |  |  |  |  |  |
| Y3ad-2 | 9.46 | 1.50 | 0.0111 |  |  |  |  |
| Y3ad2-EB | 1.45 | 0.22 | 0.1363 | 0.0119 |  |  |  |
| Y3+D166N | 0.12 | 0.03 | 0.0001 | 0.0084 | 0.0085 |  |  |
| Y3+N195K | 2.00 | 0.27 | 0.0356 | 0.0141 | 0.1730 |  |  |
| Y3+E433K | 1.14 | 0.05 | 0.0786 | 0.0115 | 0.2535 |  |  |
| Y3+D166N+N195K | 1.00 | 0.23 | 0.9958 | 0.0101 | 0.2101 |  |  |
| Y3+E433K+D166N | 1.08 | 0.08 | 0.4001 | 0.0112 | 0.1998 |  |  |
| Y3+E433K+N195K | 3.01 | 0.38 | 0.0129 | 0.0199 | 0.0169 |  |  |
| Y3+D166N+N195K+E433K | 3.73 | 0.63 | 0.0229 | 0.0242 | 0.0304 |  |  |
| Y3ad2-EC | 7.80 | 0.96 | 0.0057 | 0.3935 | 0.0054 |  |  |
|  | vs Y3+D166N | vs Y3+N195K | vs Y3+E433K | vs Y3+DN+NK | vs Y3+DN+EK | vs Y3+NK+EK | vs +DN+NK+EK |
| Y3+D166N |  |  |  |  |  |  |  |
| Y3+N195K | 0.0059 |  |  |  |  |  |  |
| Y3+E433K | 0.0000 | 0.0487 |  |  |  |  |  |
| Y3+D166N+N195K | 0.0310 | 0.0327 | 0.6028 |  |  |  |  |
| Y3+E433K+D166N | 0.0005 | 0.0388 | 0.6143 | 0.7530 |  |  |  |
| Y3+E433K+N195K | 0.0044 | 0.0773 | 0.0147 | 0.0062 | 0.0123 |  |  |
| Y3+D166N+N195K+E433K | 0.0105 | 0.0646 | 0.0258 | 0.0171 | 0.0237 | 0.3747 |  |
| Y3ad2-EC | 0.0040 | 0.0065 | 0.0060 | 0.0043 | 0.0057 | 0.0101 | 0.0153 |

**Supplementary Table 5. Mean values ± SE and *p*-values for Y3ad-2 and its E433 variant clones (Fig. 4B)**

|  | Mean | SE | vs Y3 | vs Y3ad-2 | vs Y3ad2-EB |
| --- | --- | --- | --- | --- | --- |
| Y3 | 1.00 | 0.00 |  |  |  |
| Y3ad-2 | 12.37 | 2.92 | 0.0301 |  |  |
| Y3ad2-EB | 1.55 | 0.15 | 0.0341 | 0.0341 |  |
| Y3+E433K | 0.91 | 0.04 | 0.0952 | 0.0295 | 0.0199 |
| Y3ad2-EB+E433K | 11.44 | 2.45 | 0.0236 | 0.8143 | 0.0270 |
| Y3+E433G | 0.51 | 0.06 | 0.0033 | 0.0270 | 0.0033 |
| Y3ad2-EB+E433G | 1.57 | 0.31 | 0.1668 | 0.0336 | 0.9602 |
| Y3+E433R | 5.28 | 0.56 | 0.0046 | 0.0915 | 0.0050 |
| Y3ad2-EB+E433R | 33.82 | 4.03 | 0.0039 | 0.0062 | 0.0040 |
|  | vs Y3+E433K | vs Y3ad2-EB+E433K | vs Y3+E433G | vs Y3ad2-EB+E433G | vs Y3+E433R |
| Y3+E433K |  |  |  |  |  |
| Y3ad2-EB+E433K | 0.0231 |  |  |  |  |
| Y3+E433G | 0.0018 | 0.0209 |  |  |  |
| Y3ad2-EB+E433G | 0.1258 | 0.0263 | 0.0415 |  |  |
| Y3+E433R | 0.0042 | 0.0834 | 0.0032 | 0.0026 |  |
| Y3ad2-EB+E433R | 0.0038 | 0.0052 | 0.0037 | 0.0039 | 0.0052 |

**Supplementary Table 6. *P*-values for neutralization assays (Fig. 5)**

| **PGT145** | +D166N+N195K | +D166N+N195K+E433R |
| --- | --- | --- |
| μg/mL | vs Y3 | vs Y3 |
| 0.31 | 0.7649 | 0.7689 |
| 0.63 | 0.0231 | 0.2204 |
| 1.25 | 0.4395 | 0.2492 |
| 2.50 | 0.2202 | 0.0487 |
| 5.00 | 0.3886 | 0.9583 |
| **PG16** | +D166N+N195K | +D166N+N195K+E433R |
| μg/mL | vs Y3 | vs Y3 |
| 0.31 | 0.0537 | 0.1069 |
| 0.63 | 0.0010 | 0.0104 |
| 1.25 | 0.0062 | 0.0038 |
| 2.50 | 0.0076 | 0.0134 |
| 5.00 | 0.0044 | 0.0098 |
| **VRC03** | +D166N+N195K | +D166N+N195K+E433R |
| μg/mL | vs Y3 | vs Y3 |
| 0.31 | 0.0729 | 0.0425 |
| 0.63 | 0.0523 | 0.2283 |
| 1.25 | 0.0185 | 0.1321 |
| 2.50 | 0.0184 | 0.0345 |
| 5.00 | 0.1175 | 0.0723 |
| **PGT128** | +D166N+N195K | +D166N+N195K+E433R |
| μg/mL | vs Y3 | vs Y3 |
| 0.31 | 0.1783 | 0.0008 |
| 0.63 | 0.0331 | 0.0023 |
| 1.25 | 0.0268 | 0.0034 |
| 2.50 | 0.0561 | 0.0023 |
| 5.00 | 0.0297 | 0.0106 |

**Supplementary Table 7. *P*-values (vs Y3) for sCD4 sensitivity assays (Fig. 6B)**

|  | sCD4 concentration (μg/mL) | | | |
| --- | --- | --- | --- | --- |
|  | 0.25 | 0.50 | 1.00 | 2.00 |
| +D166N+N195K | 0.0809 | 0.0385 | 0.0259 | 0.0097 |
| +E433R | 0.0023 | 0.0015 | 0.0457 | 0.0100 |
| +D166N+N195K+E433R | 0.0664 | 0.0060 | 0.0241 | 0.0115 |

**Supplementary Table 8. Binding energy between WT /Env mutant clones and CD4**

|  | Binding Energy (kcal/mol) | |
| --- | --- | --- |
|  | MM/PBSA | MM/GBSA |
| Y3 | ­20897±2821 | ­20878±2821 |
| +D166N | ­20483±2725 | ­20465±2725 |
| +N195K | ­20956±2703 | ­20939±2703 |
| +E433R | ­21149±3000 | ­21135±3000 |
| +D166N+N195K | ­21279±2862 | ­21280±2862 |
| +D166N+N195K+E433R | ­20622±2766 | ­20601±2765 |

**Supplementary Table 9. Temporal changes in the frequencies of amino acid residues at positions corresponding to the adaptive mutations D166 and E433 identified in the Y3 Env among HIV-1 subtype B sequences**

| Position  (HXB2 numbering) | Amino acids | 2009 (%)  N=698 | 2022 (%)  N=3043 |
| --- | --- | --- | --- |
|  | Asp (D) | 77.89 | 82.35 |
| 167 | Asn (N) | 16.84 | 10.92 |
|  | Gly (G) | 2.11 | 2.80 |
|  | Glu (E) | 75.93 | 75.48 |
| 429 | Lys (K) | 7.88 | 7.82 |
|  | Arg (R) | 2.87 | 4.11 |

# 3 Supplementary references

Adachi A, Gendelman HE, Koenig S, Folks T, Willey R, Rabson A, Martin MA. Production of acquired immunodeficiency syndrome-associated retrovirus in human and nonhuman cells transfected with an infectious molecular clone. J Virol. 1986 Aug;59(2):284-291. doi: 10.1128/JVI.59.2.284-291.1986.

Crooks GE, Hon G, Chandonia JM, Brenner SE. WebLogo: a sequence logo generator. Genome Res. 2004 Jun;14(6):1188-90. doi: 10.1101/gr.849004. doi:10.3389/fmicb.2011.00115.

Doi N, Fujiwara S, Adachi A, Nomaguchi M. 2011. Rhesus M1.3S cells suitable for biological evaluation of macaque-tropic HIV/SIV clones. Front Microbiol 2:115. doi:10.3389/fmicb.2011.00115.

Kamada K, Igarashi T, Martin MA, Khamsri B, Hatcho K, Yamashita T, Fujita M, Uchiyama T, Adachi A. Generation of HIV-1 derivatives that productively infect macaque monkey lymphoid cells. Proc Natl Acad Sci U S A. 2006 Nov 7;103(45):16959-16964. doi: 10.1073/pnas.0608289103.

Koma T, Odaka T, Lee S-I, Doi N, Kondo T, Okuma K, Fujisawa J-I, Adachi A, Nomaguchi M. Humanized mice generated by intra-bone marrow injection of CD133-positive hematopoietic stem cells: application to HIV-1 research. Front Virol. 2023 3:1192184 doi: 10.3389/fviro.2023.1192184.

Larsson A. AliView: a fast and lightweight alignment viewer and editor for large datasets. Bioinformatics. 2014 Nov 15;30(22):3276-8. doi: 10.1093/bioinformatics/btu531. Epub 2014 Aug 5. PMID: 25095880; PMCID: PMC4221126.

Madeira F, Madhusoodanan N, Lee J, Eusebi A, Niewielska A, Tivey ARN, Lopez R, Butcher S. The EMBL-EBI Job Dispatcher sequence analysis tools framework in 2024. Nucleic Acids Res. 2024 Jul 5;52(W1):W521-W525. doi: 10.1093/nar/gkae241.

Miller BR 3rd, McGee TD Jr, Swails JM, Homeyer N, Gohlke H, Roitberg AE. MMPBSA.py: An Efficient Program for End-State Free Energy Calculations. J Chem Theory Comput. 2012 Sep 11;8(9):3314-21. doi: 10.1021/ct300418h.

Nomaguchi M, Yokoyama M, Kono K, Nakayama EE, Shioda T, Doi N, Fujiwara S, Saito A, Akari H, Miyakawa K, Ryo A, Ode H, Iwatani Y, Miura T, Igarashi T, Sato H, Adachi A. Generation of rhesus macaque-tropic HIV-1 clones that are resistant to major anti-HIV-1 restriction factors. J Virol. 2013 Nov;87(21):11447-11461. doi: 10.1128/JVI.01549-13.

Willey RL, Smith DH, Lasky LA, Theodore TS, Earl PL, Moss B, Capon DJ, Martin MA. In vitro mutagenesis identifies a region within the envelope gene of the human immunodeficiency virus that is critical for infectivity. J Virol. 1988 Jan;62(1):139-147. doi: 10.1128/JVI.62.1.139-147.1988.
